# Analysis of the sulfate permease family in *Bursaphelenchus xylophilus* in the nematode development and stress adaptation

**DOI:** 10.3389/fpls.2025.1630288

**Published:** 2025-10-13

**Authors:** Haixiang Li, Rui Wang, Na Pu, Song Yang, Jie Chen, Xin Hao

**Affiliations:** ^1^ The Key Laboratory of Forest Resources Conservation and Utilization in the Southwest Mountains of China Ministry of Education, Southwest Forestry University, Kunming, China; ^2^ The Construction Program of Yunnan Provincial Key Laboratory for Conservation and Utilization of In-forest Resource, Southwest Forestry University, Kunming, China

**Keywords:** *Bursaphelenchus xylophilus*, sulfate permease family, gene and protein structure, bioinformatic analysis, gene expression

## Abstract

**Introduction:**

Pine wilt disease (PWD), caused by the pine wood nematode (PWN) *Bursaphelenchus xylophilus*, poses a significant threat to global pine forests. The sulfate permease (SULP) family is essential for sulfate transport, sulfur assimilation and cellular homeostasis, yet it remains uncharacterized in *B. xylophilus*. This study aimed to comprehensively identify all members of the SULP family in *B. xylophilus* and to elucidate their roles in nematode development and stress adaptation.

**Methods:**

Through genomic data analysis, we identified 10 members of the SULP family in *B. xylophilus* and conducted a comprehensive characterization of their physicochemical properties, conserved motifs, protein structures, and gene expression profiles across different developmental stages.

**Results:**

The results revealed Bx-sulps were located on 5 chromosomes of *B. xylophilus*. Phylogenetic analysis unveiled both conserved and divergent evolutionary patterns of these proteins compared to counterparts in other nematodes. Expression analysis demonstrated upregulation of *Bx-sulps* during the dauer third-instar larva (D3) stage, suggesting their involvement in stress response and diapause. Moreover, certain Bx-sulps exhibited high expression levels in adult stages, indicating a potential role in reproductive processes.

**Discussion:**

The study presents the first comprehensive examination of BxSULP family, shed light on its significance in nematode development and stress adaptation. These findings provide the groundwork for further functional investigations and may aid in the development of targeted strategies for managing PWD.

## 1 Introduction

Pine wilt disease (PWD), caused by the pine wood nematode (PWN) *Bursaphelenchus xylophilus*, is one of the greatest threats to pine (Yu et al., 2025). First identified in Japan in the 20th century, PWD has become a major concern for global forestry (Mamiya, 1983). In China, PWD has impacted 620 counties in 18 provinces, spreading rapidly to westward and northward (Chen et al., 2022; Li et al., 2025). At present, the management strategies for PWD focus on integration monitoring, early detection, and rapid response methods. Integrated pest management (IPM) practices, including cultural techniques (such as proper watering, mulching, and pruning to enhance tree health), biological control, and chemical interventions (insecticides and nematicides), are commonly employed to reduce PWD incidence (Ahmed et al., 2025). *B. xylophilus* is primarily spread by vector insects, notably the genus *Monochamus*. These insects introduce *B. xylophilus* into healthy pines while laying eggs or feeding on infected (Togashi et al., 2019).

Under natural conditions, *B. xylophilus* alternates between two life-history modules: a propagative phase within living pines and a dispersal phase between hosts (Kang et al., 2009). During propagation, eggs hatched into second instar larva (L2), which molt through third instar larva (L3) and fourth instar larva (L4) before maturing into adults (Tanaka et al., 2019). Entirely confined to the resin canal system, this phase supported exponential population growth under favorable temperature and moisture. Declining temperature and host humidity triggered L2 to divert into the dispersal pathway, molting into dauer third-instar larva (D3) (Hoover et al., 2010). These accumulate lipid reserves, thicken their cuticles, and aggregated around pupal chambers of the *M. alternatus* (Tian et al., 2022). After quiescence, D3 molted into dauer fourth-instar larva (D4), which were transported by emerging beetles and inoculated into new host trees during maturation feeding, initiating further infections (Mamiya, 1983).

*B. xylophilus* is capable of proliferating and multiplying within the resin canals of its pine host, a trait closely linked to the adaptive evolution of its gene family (Zhang et al., 2020). Investigating the expression patterns and roles of individual gene family members of *B. xylophilus* can elucidate their contributions to the nematode’s growth and development, offering valuable insights for identifying molecular targets to manage *B. xylophilus*. Research of gene families in *B. xylophilus* has advanced considerably in recent decades. Genome analysis has revealed several gene families crucial for the adaptive evolution and pathogenicity of *B. xylophilus*. The cytochrome P450 (CYP450) family in *B. xylophilus* has undergone significant amplification, facilitating the detoxification and metabolism of pine defense chemicals by the nematode (Liu et al., 2025). The flavin-containing monooxygenase (FMO) family contributes to detoxification processes, enabling the nematode to thrive in pine resin channels (Bailleul et al., 2023). The glycoside hydrolase (GH) family aids in the degradation of pine cell walls, thereby enhancing the nematode’s pathogenicity (Shinya et al., 2021). Nevertheless, investigations into the Sulfate permease family in *B. xylophilus* remain relatively limited. Sulfur is essential for synthesizing sulfur-containing amino acids like cysteine and methionine, crucial for protein synthesis in nematodes (Ali and Nozaki, 2007). This suggested that *Bx-sulp*s might similarly determine PWN fitness within the host, making them high-value targets for RNAi- or HIGS-based control strategies.

In *B. xylophilus*, the SULP family may be linked to the evolutionary adaptation of nematodes to pine environments. Through enhanced sulfate uptake and utilization, *B. xylophilus* may exhibit improved adaptation to pine defense mechanisms. To date, the functional characterization of the SULP family in *B. xylophilus* remains unexplored. This study represents the first systematic investigation into the SULP family within *B. xylophilus*. The research aimed to identify all members of the SULP family in *B. xylophilus* and to elucidate their roles in nematode development and stress adaptation.

## 2 Materials and methods

### 2.1 Physicochemical properties analysis of BxSULPs

Firstly, we obtained the whole genome of *B. xylophilus* (SAMEA7282713) (https://www.ncbi.nlm.nih.gov/datasets/genome/GCA_904067135.1/) data from Wormbase (https://parasite.wormbase.org). To obtain a representative set of SULP orthologues, we first screened all publicly available nematode genomes deposited in NCBI database (https://www.ncbi.nlm.nih.gov/gene) (Howe et al., 2016) for the presence of complete, well-annotated SULP families. After applying strict criteria for sequence completeness and annotation quality, only three genomes (*Brugia malayi*, *Caenorhabditis elegans*, and *Caenorhabditis briggsae*) were retained. We used BLASTX to compare the queried protein sequence with the protein database of *B. xylophilus* to obtain the candidate BxSULPs sequence. BLAST searches were performed with an E-value cut-off of 1 × 10^-5^, ≥ 30% query coverage and ≥ 25% identity. After that, we queried the Pfam database (https://pfam.xfam.org/) to search for the conserved structural domains possessed by the SULP protein and downloaded its Hidden Markov Model (El-Gebali et al., 2019). The protein sequences with the Hidden Markov Model were then searched from the protein database of *B. xylophilus* by HMMER3.0 software. HMMER 3.0 was run using the trusted cut-off (TC) defined in Pfam for the Sulfate_transp and STAS domains, with an inclusion threshold of E ≤ 1 × 10^–5^ and a minimum alignment coverage of 70% of the Pfam model. Finally, the candidate sequences were characterized using NCBI-conserved domains (https://www.ncbi.nlm.nih.gov/Structure/cdd) (Lu et al., 2020b) and InterPro (https://www.ebi.ac.uk/Interpro) (Cantelli et al., 2021) to eliminate duplicates and acquire the protein sequences of BxSULPs. The physicochemical properties of BxSULPs were analyzed using Expasy 3.0 (https://web.expasy.org/protparam) (Duvaud et al., 2021).

### 2.2 Phylogenetic analysis of BxSULPs and analysis of conserved motifs

In order to evaluate the extent of phylogenetic similarity among the 10 protein sequences, we conducted phylogenetic and conserved motif analyses of BxSULPs. Evolutionary relationships of BxSULP were analyzed using MEGA 11.0 software through multiple sequence alignments of 10 BxSULPs and the optimal model for the maximum likelihood tree was determined. Based on the prediction results of the maximum likelihood tree in the best model, the LG model was selected to construct the phylogenetic evolutionary tree of BxSULP. This model had a BIC (Bayesian Information Criterion) value of 11645.256 and an AICc (Corrected Akaike Information Criterion) value of 11362.515. The model was chosen because it provided the lowest BIC value (11645.256), indicating that it is the best fit for the data among the tested models (Luo et al., 2010). A maximum likelihood evolutionary tree was constructed with the Bootstrap test performed 1000 times (Koichiro et al., 2021). Subsequently, the constructed phylogenetic evolutionary tree was landscaped using iTOL (https://itol.embl.de/tree/) (Letunic and Bork, 2024). Gene information for *Bx-sulp*s was obtained from the gene structure annotation file, and gene structure analysis was conducted using the GSDS 2.0 (http://gsds.gao-lab.org/) (Hu et al., 2015). Additionally, the distribution of conserved motifs in BxSULPs was examined using MEME Suite 5.5.6 (https://meme-suite.org/meme/) (Bailey et al., 2015) with default parameters and a motif value of 8. The results from MEME were combined with the maximum likelihood tree to create gene structure maps with annotated conserved motifs.

### 2.3 Chromosomal distribution and protein structure analysis of BxSULPs

The gene density information of *B. xylophilus* from the annotation file of gene structure was extracted by Gene Density Profile function of TBtools. Then, we integrated the annotation file and the density file to map the distribution of BxSULP on chromosomes by Gene Location Visualize from GTF/GFF function (Chen et al., 2020, 2023). Subsequent analysis included predicting the secondary structure of BxSULPs using online tools like Prabi (https://doua.prabi.fr/software/cap3) (Thibaut et al., 2015), NetPhos 3.1 (https://services.healthtech.dtu.dk/services/NetPhos-3.1/) (Blom et al., 1999), and Protter 1.0 (http://wlab.ethz.ch/protter/start/) (Ulrich et al., 2014). Swiss-Model (https://swissmodel.expasy.org/interactive) (Marco et al., 2014) was employed to forecast the protein tertiary structure, and SAVES v6.1 (https://saves.mbi.ucla.edu) was used to create Ramachandran diagrams.

### 2.4 Isolation and identification of *B. xylophilus*

*B. xylophilus* was isolated from symptomatic *Pinus thunbergii* logs collected in Zhejiang Province, China. Xylem samples were cut into 2–3 cm thick disks and placed on a modified Baermann funnel (25 °C, 24 h) (Shinya et al., 2013). Nematodes that migrated into the collection tube were recovered and examined under a light microscope. Individual adult females were carefully picked out and identified to species according to the morphological diagnostic criteria (Abelleira et al., 2011). Only those specimens that exhibit the typical morphological characteristics of *B. xylophilus* are retained for subsequent cultures and experiments.

### 2.5 Culture of *B. xylophilus* in different developmental stages

Synchronized eggs were obtained from embryos by incubating mixed-stage *B. xylophilus* in petri dishes at 25 °C in the absence of light for 10 minutes. This process facilitated the adhesion of eggs to the substrate through surface glycoproteins. The top layer containing water and nematodes was then removed to harvest the synchronized eggs. Subsequently, these eggs were allowed to hatch at 25 °C in the dark to yield second instar larvae (L2) in a nutrient-free setting. These synchronized L2 larvae were then transferred onto a *Botrytis cinerea* lawn on a PDA plate. Nematodes were harvested at 24, 48, and 72-hour intervals to obtain third instar larvae (L3), fourth instar larvae (L4), and adults, respectively. Dauer third-instar larvae (D3) and dauer fourth-instar larvae (D4) were isolated from infected pinewood by Baermann funnel. Male and female nematodes were manually sorted under microscope using worm pickers (Tang et al., 2020).

### 2.6 Expression profiles of BxSULP family in different developmental stages

Gene expression in distinct developmental stages was analyzed by reverse-transcription quantitative polymerase chain reaction (RT-qPCR) by synchronizing *B. xylophilus* in different developmental stages. To explore the molecular response mechanisms of *Bx-sulp*s to various treatments, expression levels of 10 *Bx-sulp*s were determined across various developmental stages of *B. xylophilus*, including eggs. The statistical significance of the data was determined using a one-way analysis of variance (ANOVA) using GraphPad Prism 10.6.0.890 with a student’s *t*-test with *p* < 0.05 as the significance threshold.

### 2.7 Validation of gene expression by RT-qPCR

Differentially expressed genes were chosen for validation via RT-qPCR (Tanaka et al., 2019). Primer sequences were generated with Primer 6.0 software and subsequently synthesized by Sangon Biotech (Shanghai, China) (**Table 1**). Amplification was performed on a QuantStudio 1 Plus instrument (Thermo Fisher Scientific, USA) following the manufacturer’s instructions for the One Step PrimeScript™ RT-PCR Kit (RR064A, Beijing), and relative gene expression was calculated using the -ΔΔCt method. Gene-expression data were normalized to the Bx28S rRNA reference gene.

**Table 1:** RT-qPCR primer sequences.

Protein ID of *B. xylophilus*	Protein name	Primer sequences
BXYJ5.010268100	BxSULP1	F: CCCACTTTCAGGCTCTCCAG R: CCGAAACGACAGATTGGTGC
BXYJ5.020105600	BxSULP2	F: CTGGTTTCATTAACGGCGGC R: ACACCGTTAAGAGCAGGGTG
BXYJ5.020147800	BxSULP3	F: AACTTTTCGCTGTCTCCGCT R: AGACGGCGATGTGTTCTGAT
BXYJ5.030165800	BxSULP4	F: CACGCTTTCGAGCTATGTGC R: GAGACCGCCAATACTCTGGG
BXYJ5.030215700	BxSULP5	F: GGCCAAAATTCCAGCACCTG R: CCAGCAGATCCGAGCCATAG
BXYJ5.050106800	BxSULP6	F: CTTGGCTTCATGGGGACACT R: GACTCCACACTCAACGCTCA
BXYJ5.050140100	BxSULP7	F: AACTGCCCTGATGGTTGGAG R: CGGGGGCTACATCCATTCTC
BXYJ5.050219300	BxSULP8	F: TTTTGTCGCACCTGACCGTA R: GTCAATTCGTATACACTTTTTCCGT
BXYJ5.060009300	BxSULP9	F: TGATAGGGAAAGAGGGGCGA R: GCCAGAACGGAGTAGGCAAT
BXYJ5.060037700	BxSULP10	F: GAAGCCACTGCCAAACCAAG R: TCATCGGGTGGGAGTCTGAT
28S	/	F: AACCGAACACGCGACAATAG R: GTGCGTATTCAGCCTTCTGG

## 3 Results

### 3.1 Identification of BxSULPs

Following a BLASTX search in the Wormbase database and subsequent validation of conserved domains using Conserved Domains and InterPro, we identified 10 distinct SULP proteins in *B. xylophilus*, designated as BxSULP1 to BxSULP10 based according to their ascending order on chromosomes (**Table 2**). To further elucidate the evolutionary relationships, we conducted a comparative analysis of the protein sequences of SULP found in BxSULP1 to BxSULP10 with those of analogous proteins from other nematodes.

**Table 2:** Comparison of BxSULPs with other nematodes.

Protein ID of *B. xylophilus*	Protein name	Sequence ID in NCBI	Protein name in other nematodes	Other nematodes name	Scores of blastP	Similarity *e*-value	% identity
BXYJ5.010268100	BxSULP1	CBG_23242	Protein CBR-SULP-4	*Caenorhabditis briggsae*	335	1.8×10^-121^	36.2
BXYJ5.020105600	BxSULP2	CELE_F41D9.5	Protein CE-SULP-3	*Caenorhabditis elegans*	302	1.5×10^-140^	58.4
BXYJ5.020147800	BxSULP3	CELE_ZK287.2	Protein CE-SULP-8	*Caenorhabditis elegans*	199	8.7×10^-62^	35.0
BXYJ5.030165800	BxSULP4	CBG_23242	Protein CBR-SULP-4	*Caenorhabditis briggsae*	224	2.6×10^-37^	28.4
BXYJ5.030215700	BxSULP5	CELE_K12G11.2	Protein CE-SULP-5	*Caenorhabditis elegans*	147	2.3×10^-37^	28.3
BXYJ5.050106800	BxSULP6	CBG_23241	Protein CBR-SULP-5	*Caenorhabditis briggsae*	234	2.2×10^-101^	47.0
BXYJ5.050140100	BxSULP7	CELE_W01B11.2	Protein CE-SULP-6	*Caenorhabditis elegans*	249	2.5×10^-142^	46.7
BXYJ5.050219300	BxSULP8	CELE_ZK287.2	Protein CE-SULP-8	*Caenorhabditis briggsae*	462	8.4×10^-148^	56.5
BXYJ5.060009300	BxSULP9	CBG_23241	Protein CBR-SULP-5	*Caenorhabditis elegans*	343	3.1×10^-133^	51.5
BXYJ5.060037700	BxSULP10	CBG_23241	Protein CBR-SULP-5	*Caenorhabditis briggsae*	303	1.9×10^-129^	48.9

Comparative analysis of BxSULPs with homologs from nematodes like *C. briggsae* and *C. elegans* revealed both conserved and divergent evolutionary patterns. High BLASTP scores (above 100) and low E-values (below 1×10^-10^) indicated significant sequence similarity, implying shared conserved structural and functional domains, notably in the Sulfate Transporter and Anti-sigma factor antagonist (STAS) domain crucial for sulfate binding and transport. Notably, BxSULP8 exhibited a remarkable BLASTP score of 462 and an exceptionally low E-value of 8.4×10^–148^ compared to a STAS domain-containing protein in *C. elegans*. This high degree of sequence similarity suggested that BxSULP8 was highly conserved and likely played a crucial role in sulfate transport, similar to its orthologs in other nematodes. Given the critical function of the STAS domain in sulfate binding and transport, BxSULP8 might be particularly important for maintaining sulfate homeostasis in *B. xylophilus*. This warrants further functional investigations to elucidate its specific role in nematode biology. These differences underscored the importance of understanding the evolutionary and functional contexts of SULP across diverse nematode species, providing insights for targeted potential control strategies and targeted interventions.

### 3.2 Physicochemical analysis of BxSULPs

The average number of amino acids in BxSULPs was 202.2, with an average molecular mass of 22.71 kDa. The size of BxSULP varied, ranging from 17.63 kDa (BxSULP1) to 29.47 kDa (BxSULP2). Notably, BxSULP10, the largest protein, may exhibit more intricate functionalities or interactions. The isoelectric points (pI) of BxSULPs spanned from 6.51 (BxSULP6) to 10.19 (BxSULP9). Proteins with pI values near neutrality are anticipated to demonstrate enhanced stability under physiological conditions, whereas those with extreme pI values might serve specialized roles. BxSULP9 could participate in processes occurring in high pH environments. BxSULP6, characterized by the highest aliphatic index (119.51), may exhibit greater resistance to thermal denaturation compared to other BxSULPs, a critical feature for its potential function in extreme nematode environments. BxSULP2, BxSULP5, BxSULP9, and BxSULP10 were hydrophilic, while the remaining proteins were hydrophobic. BxSULP4, BxSULP7, and BxSULP8 demonstrated stability, contrasting with the instability observed in the other proteins. This suggested that protein may possess more robust structures, potentially ensuring sustained functionality over time (**Table 3**).

**Table 3:** Physicochemical properties analysis of BxSULPs.

Protein name	Number of amino acids	Molecular weight/kDa	Isoelectric point^1^	Aliphatic index^2^	Hydropathicity^3^	Instability index^4^	Stability
BxSULP1	158	17.63	9.96	85.19	0.068	51.61	Unstable
BxSULP2	257	29.47	9.60	94.79	-0.125	46.28	Unstable
BxSULP3	200	22.32	9.30	99.95	0.402	46.92	Unstable
BxSULP4	167	18.75	8.58	105.03	0.100	34.51	Stable
BxSULP5	173	20.23	10.09	82.89	-0.124	53.37	Unstable
BxSULP6	225	25.52	6.51	119.51	0.510	43.38	Unstable
BxSULP7	181	19.76	8.22	114.64	0.893	37.56	Stable
BxSULP8	227	24.71	9.90	109.91	0.763	31.44	Stable
BxSULP9	172	19.50	10.19	72.67	-0.247	59.18	Unstable
BxSULP10	262	29.18	7.74	75.92	-0.049	49.91	Unstable

^1^ Isoelectric point (pI): The isoelectric point (pI) is the pH at which a protein carries no net electrical charge.

^2^ Aliphatic indexes: The Aliphatic Index is a measure that describes the relative volume occupied by aliphatic side chains in a protein.

^3^ Hydropathicity: Hydropathicity is a relative value used to measure the hydrophobicity or hydrophilicity of a molecule. Positive values indicate hydrophobicity, while negative values indicate hydrophilicity.

^4^ Instability index: The Instability Index is a metric used to evaluate the stability of proteins. If the Instability Index is less than 40, the protein is considered stable. If the Instability Index is greater than 40, the protein is unstable.

### 3.3 Phylogenetic and conserved motif analysis of BxSULPs

**Figure 1:**
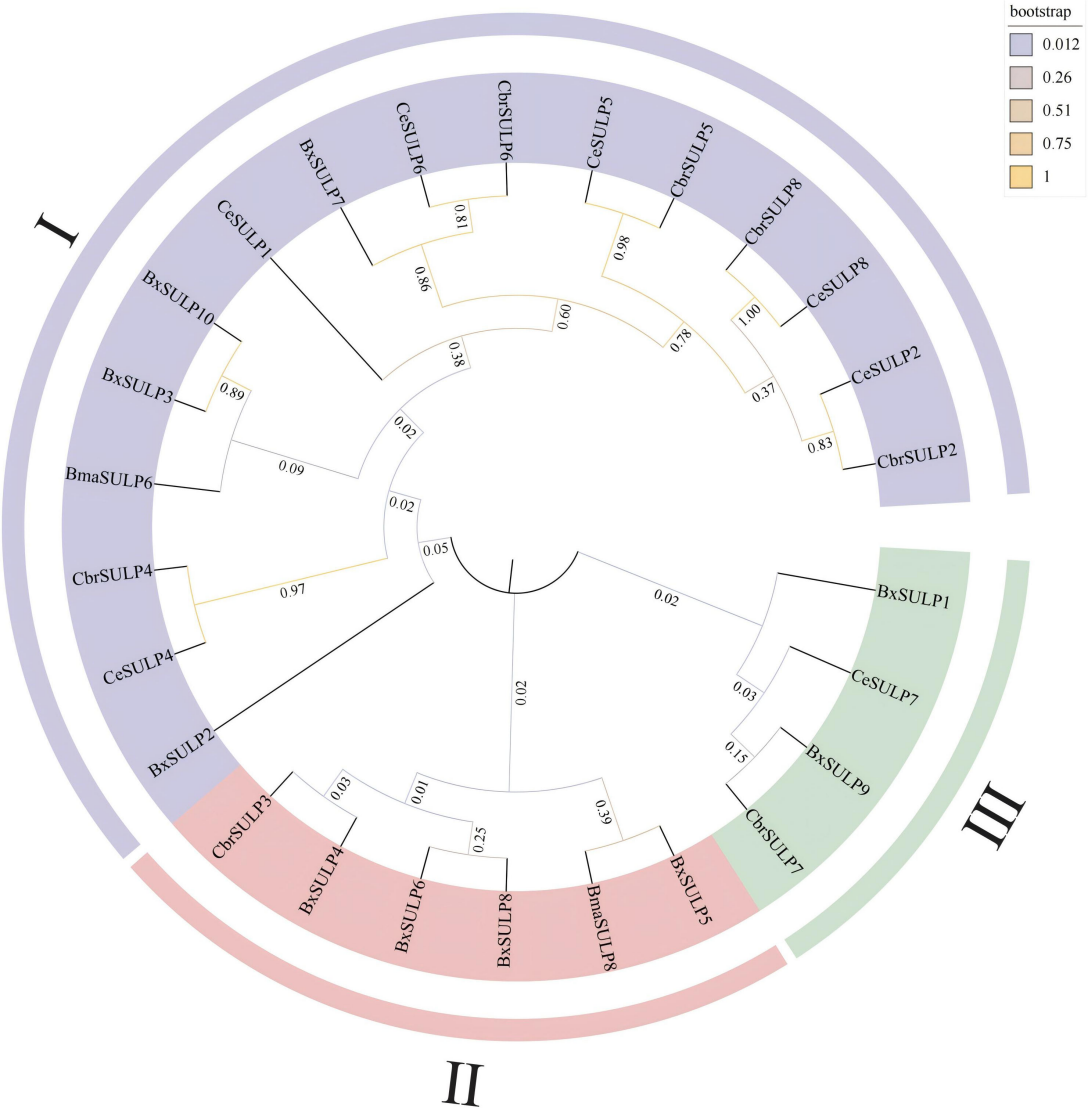
Phylogenetic tree of BxSULPs and SULPs in other nematodes. Bx represents *Bursaphelenchus xylophilus*, Bma represents *Brugia malayi*, Cbr represents *Caenorhabditis briggsae* and Ce represents *Caenorhabditis elegans*.

We performed phylogenetic analysis on the full-length protein sequences. The conserved STAS and Sulfate_transp domains were subsequently mapped onto the alignment to verify motif conservation. The phylogenetic analysis revealed distinct clustering patterns among SULPs, indicating conserved and divergent evolutionary trajectories. The grouping of BxSULPs with homologs from closely related nematodes like *C. briggsae* and *C. elegans* suggested significant sequence conservation and shared ancestry. For example, BxSULP7 clustered closely with CbrSULP6 and CeSULP6, implying a strong evolutionary relationship, while functional equivalence remains to be experimentally determined. However, notable divergence was observed among SULPs from different nematode species. BxSULP2 exhibited a unique branching pattern, suggesting potential functional or adaptive divergence specific to *B. xylophilus*, possibly linked to its distinct ecological niche. The phylogenetic tree categorized BxSULP into distinct clades, denoted by different colors. Purple represents the SULP I family, red the SULP II family, and green the SULP III family, highlighting separate evolutionary lineages within the SULP family. SULP4, SULP5, SULP6, SULP8 of *B. xylophilus*, SULP3 of *C. briggsae* and SULP8 of *B. malayi* form a well-supported clade. Similarly, SULP1, SULP9 of *B. xylophilus*, SULP7 of *C. elegans* and SULP7 of *C. briggsae* clustered together in another distinct clade. SULP3, SULP7, SULP10 of *B. xylophilus*, SULP1, SULP2, SULP4, SULP5, SULP6, SULP8 of *C. elegans*, SULP2, SULP4, SULP5, SULP6, SULP8 of *C. briggsae* and SULP6 of *B. malayi* clustered in a separate branch, supporting the concept of gene duplication and subsequent functional diversification. These clades were also strongly supported by high bootstrap values, affirming their robustness (**Figure 1**).

In the context of sulfate permeases, the motifs are likely involved in critical functions such as sulfate binding, transmembrane transport, and protein stability (**Supplementary Figure S1**). Analysis determined the compositions of introns and exons in *Bxsulp*s, revealing that the number of exons ranged from 7 to 16 across the 10 genes. Specifically, BxSULP3, BxSULP8 and BxSULP10 had only 7 exons, while BxSULP5 exhibited the highest number with 14 exons. Examination of structural motifs in the BxSULPs unveiled shared motifs among these proteins, as corroborated by the analysis of conserved motif structures (**Supplementary Figure S2**). The figure illustrated the conserved motifs identified within BxSULPs, emphasizing regions of sequence similarity across the proteins. These motifs are short, conserved sequences that are likely involved in critical functions such as sulfate binding, transmembrane transport, and protein stability. The clustering of these motifs indicates regions of high conservation, suggesting potential functional roles.

### 3.4 Chromosomal distribution of *Bx-sulp*s

**Figure 2:**
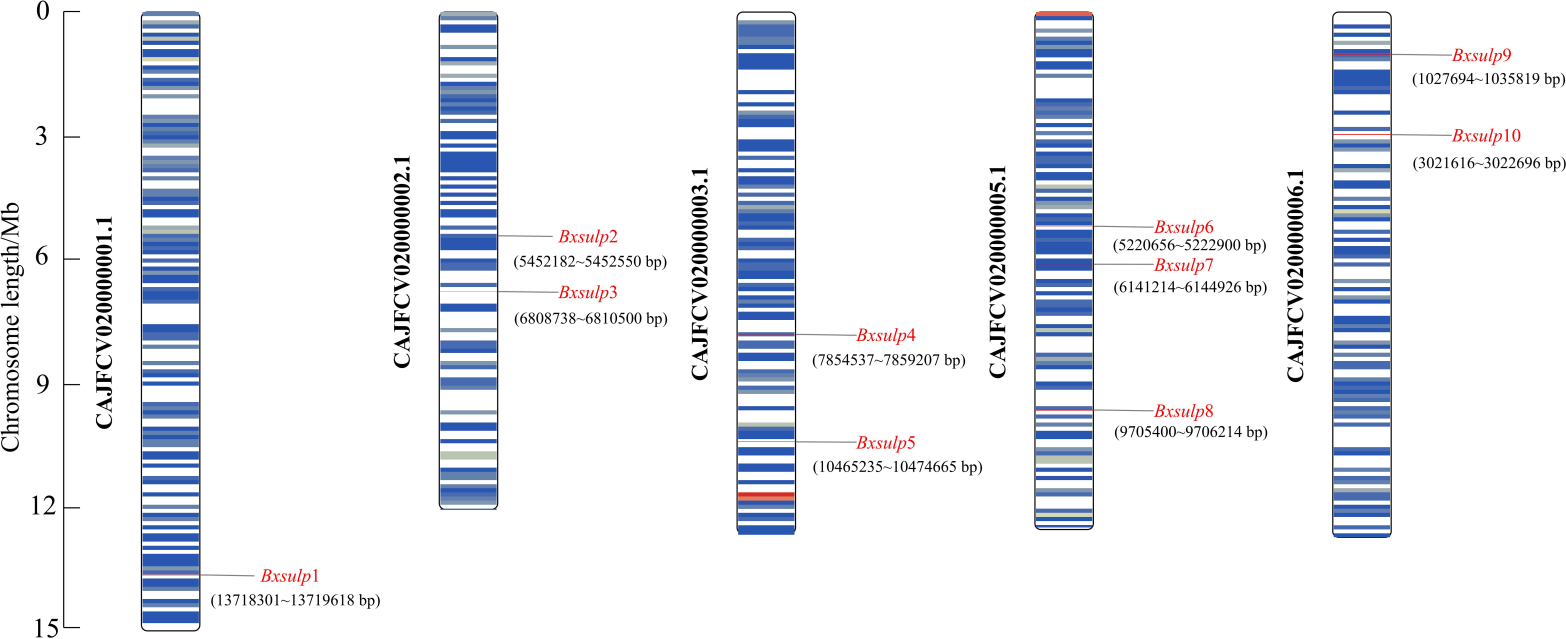
Chromosomal position of *Bx-sulp*s. Note: Each column represents a different chromosome, labeled from 1 to 5. The vertical axis represents the relative positions of genes along the chromosomes, with 5’ and 3’ regions indicated.

The chromosomal localization of the 10 *Bx-sulp*s in *B. xylophilus* was determined using TBtools v2.056, precisely mapping the locations of these channel-encoding genes across various chromosomes (**Figure 2**). Analysis of chromosomal localization revealed that the genes encoding the 10 SULP proteins were distributed among five chromosomes of *B. xylophilus*. Specifically, on chromosome 1, *Bx-sulp*1 was situated in the 3′ region. *Bx-sulp*2 and *Bx-sulp*3 were positioned in the middle of chromosome 2. Furthermore, *Bx-sulp*4 and *Bx-sulp*5 were identified in the 3′ region of chromosome 3. Within chromosome 5, *Bx-sulp*6 and *Bx-sulp*7 were located in the central region, and *Bx-sulp*8 was found in the 3′ region. Additionally, *Bx-sulp*9 and *Bx-sulp*10 were situated in the 5′ region of chromosome 6, with no genes present on chromosome 4. The dispersion of *Bx-sulp*s across multiple chromosomes indicates their non-clustered distribution throughout the genome. This dispersion had implications for gene regulation, as genes located on distinct chromosomes could be governed by diverse regulatory mechanisms and environmental factors.

### 3.5 Protein structure analysis of BxSULPs

**Figure 3:**
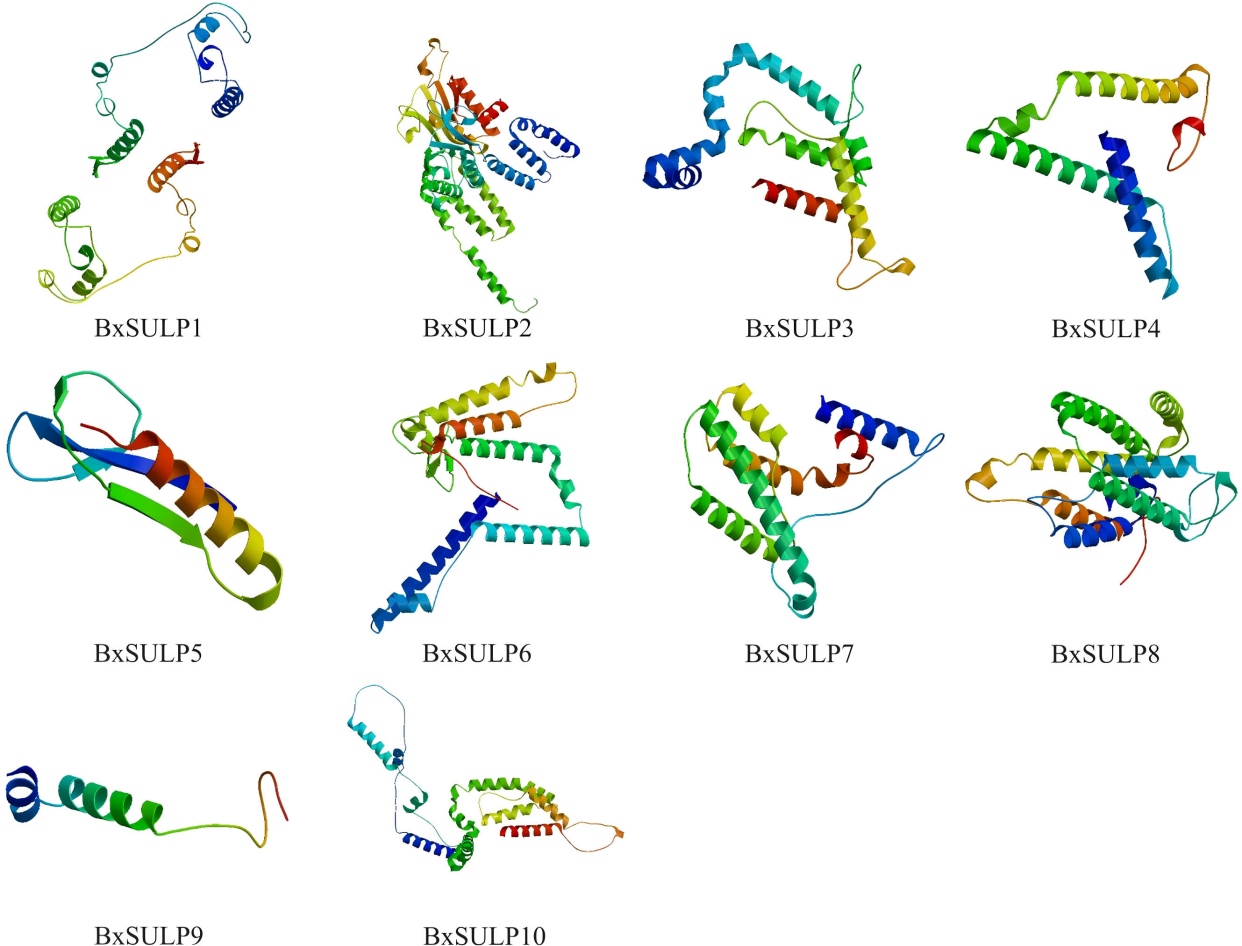
Predicted three-dimensional structures of BxSULPs, showed *α*-helices, *β*-folded structures, *β*-turns, and irregular(random) coils.

**Figure 4:**
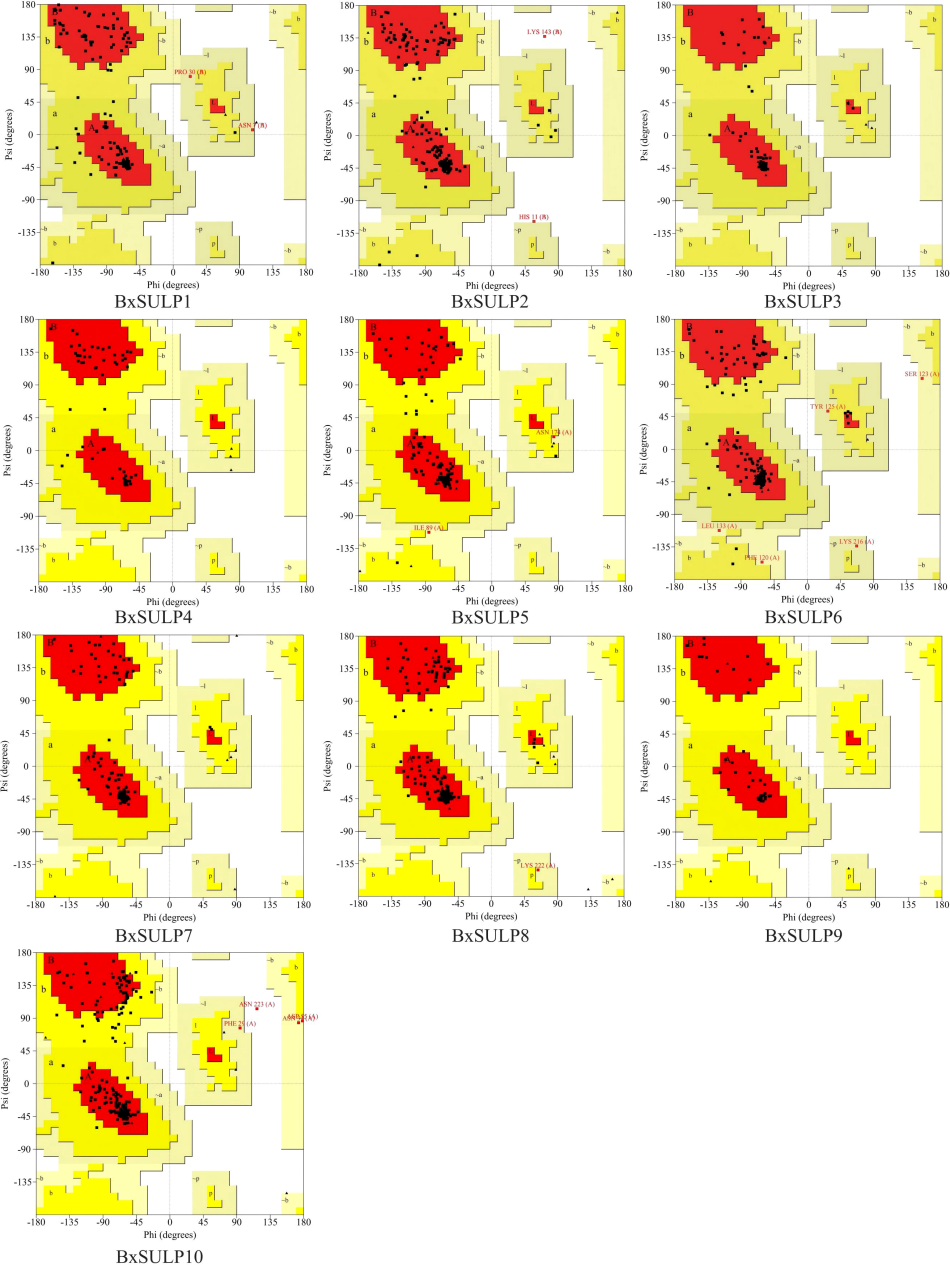
Ramachandran plot of 10 BxSULPs. The plot shows the distribution of phi (Φ) and psi (Ψ) angles for the amino acid residues in the proteins. The core regions (yellow) indicate highly stable conformations, while the allowed regions (white) show less stable but still plausible conformations.

Analysis of the three-dimensional structures of 10 BxSULPs revealed similar proportions of structural elements, including *α*-helices (32.56%–60.22%), *β*-folds (6.11%–23.12%), *β*-turns (0.60%–11.05%), and random coils (28.73%–53.82%). The transmembrane segment of BxSULPs predominantly comprised *α*-helices, facilitating their integration into the cell membrane to form channel structures for sulfate transport. Despite variations in the number and positioning of transmembrane segments across different proteins, a general conservation of these regions was observed. While BxSULPs exhibited sequence diversity, their three-dimensional structures displayed substantial similarity, indicating the preservation of core functional features essential for sulfate transport (**Table 4**; **Figure 3**). The Ramachandran plot was used to assess the three-dimensional structures of 10 proteins. The dihedral angles of BxSULPs residues were found to be located in the yellow favored regions, and the spatial structure was found to be over 90% stable, indicating a high degree of confidence in the tertiary structure. The Ramachandran plot analysis confirms the high stability of the three-dimensional structures of BxSULPs, with the majority of residues falling within the core regions, indicating a high degree of confidence in the predicted protein conformations (**Figure 4**).

**Table 4:** Secondary structure motifs of BxSULPs.

Protein ID	Number of N-glycosylation sites	Number of amino acids						Percentage of number (%)				
		T	S	Y^1^	*α*-helix^2^	*β*-fold^3^	*β*-turn^4^	Random coil^5^	*α*-helix	*β*-fold	*β*-turn	Random coil
BxSULP1	3	5	14	0	78	22	11	47	49.37	13.92	6.96	29.75
BxSULP2	1	9	17	2	107	46	9	95	41.63	17.90	3.50	36.96
BxSULP3	3	4	13	1	95	15	7	83	47.50	7.50	3.50	41.50
BxSULP4	0	4	13	3	90	26	1	50	53.89	15.57	0.60	29.94
BxSULP5	1	2	15	3	67	40	9	57	38.73	23.12	5.20	32.95
BxSULP6	2	5	6	6	109	25	5	86	48.44	11.11	2.22	38.22
BxSULP7	1	4	18	0	109	15	5	52	60.22	8.29	2.76	28.73
BxSULP8	1	7	7	2	112	32	5	78	49.34	14.10	2.20	34.36
BxSULP9	1	10	11	2	56	37	19	60	32.56	21.51	11.05	34.88
BxSULP10	2	9	12	5	98	16	7	141	37.40	6.11	2.67	53.82

^1^ T, S, Y: T is Threonine, S is Serine, and Y is Tyrosine, quantity share is the number of amino acids that make up each secondary structure as a percentage of the total number of amino acids.

^2^ α-helix: The α-helix is a common secondary structure in proteins, formed by the right-handed coiling of a polypeptide chain around a central axis.

^3^ β-fold: The β-fold is a secondary structure formed by the parallel or antiparallel alignment of polypeptide chains.

^4^ β-turn: The β-turn is a common type of turn structure, typically composed of four amino acid residues.

^5^ Random coils: Random coil refers to the parts of a protein that cannot be classified as α-helices, β-fold, or other regular secondary structures.

**Figure 5:**
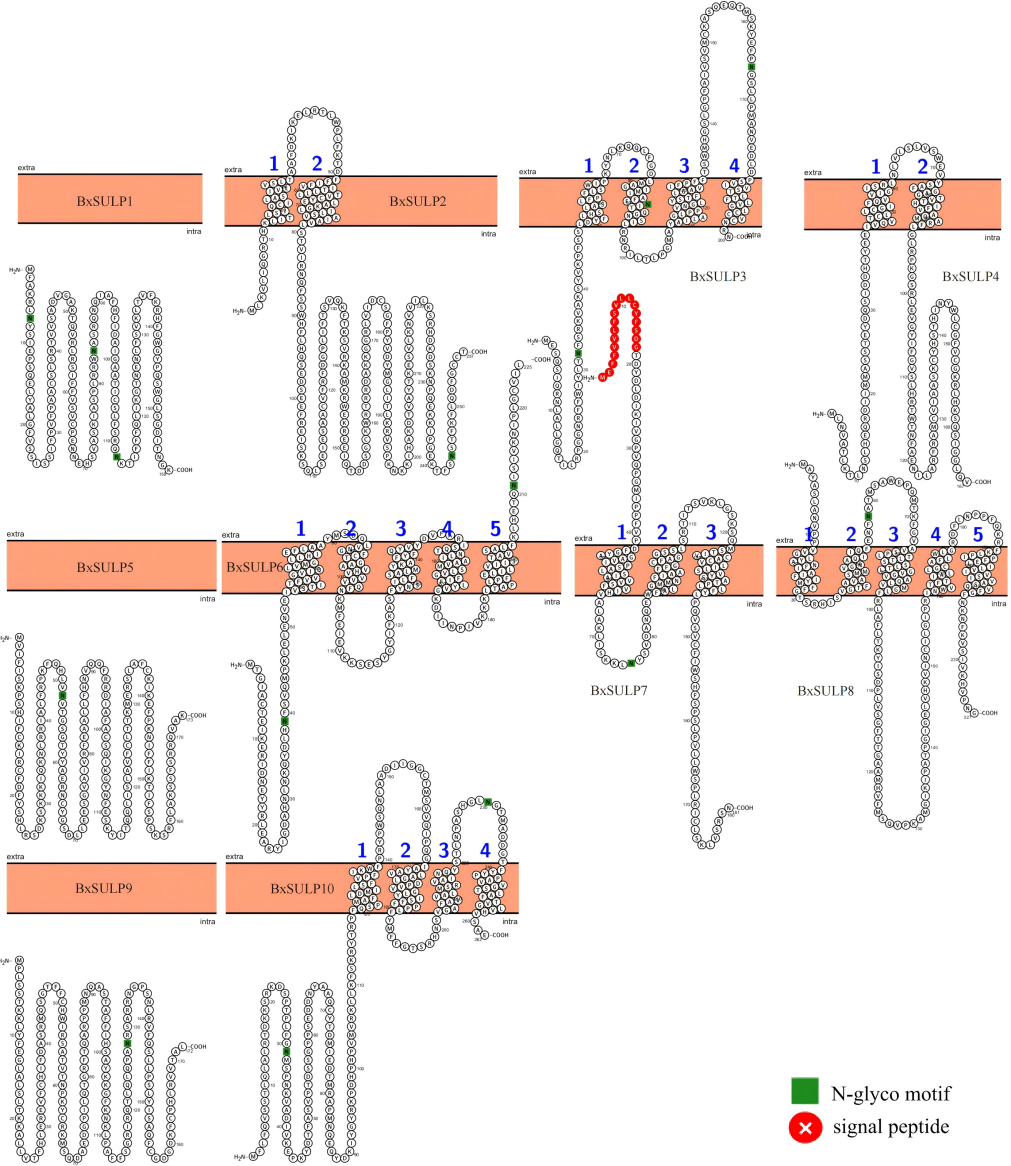
Predicted topologies of 10 BxSULPs. Glycosylation sites are indicated in green, the cell membrane is shown in orange, and signal peptides are highlighted in red.

The topological structure analysis indicated that BxSULP2, BxSULP3, BxSULP4, BxSULP6, BxSULP7, BxSULP8, and BxSULP10 were transmembrane proteins characterized by the presence of transmembrane domains enabling their integration into the cell membrane, primarily facilitating sulfate transmembrane transport. Conversely, BxSULP1, BxSULP5, and BxSULP9 were intra-membrane proteins. Although these proteins lacked transmembrane domains, they can be secreted outside the cell via signaling peptides. These signal peptides were short, hydrophobic regions at the N-terminus of the protein that guided the protein through the endoplasmic reticulum and into the extracellular space. These intra-membrane proteins operate within the extracellular matrix or engage in intercellular signaling upon secretion. Once secreted, these proteins can interact with other molecules in the extracellular matrix or participate in intercellular signaling pathways. Their functions might include binding to extracellular ligands, modulating cell-cell interactions, or influencing the local environment around the cell. Notably, BxSULP1 and BxSULP3 exhibited the highest number of N-glycosylation sites, with 3 sites each, enhancing their stability and functionality in membrane-associated or secreted contexts. This suggested a pivotal role in sulfate transport and interaction with the extracellular environment. The elongated signal peptide of BxSULP7 suggested more precise localization within the cell membrane, thereby enhancing its efficacy in sulfate transport. Despite variations in transmembrane structure, glycosylation patterns, and signal peptides among the BxSULPs, they exhibited a degree of structural similarity. These structural variations suggested functional partitioning. The multi-pass trans-membrane proteins were predicted to form the core sulfate-transport channels. In contrast, the secreted is formed that contain signal peptides might function as extracellular sulfate-binding or sulfate-donating molecules, modulate local sulfur availability or serving as signaling ligands during host invasion and diapause (**Figure 5**).

### 3.6 Expression profiles of *Bx-sulp*s in different stages

**Figure 6:**
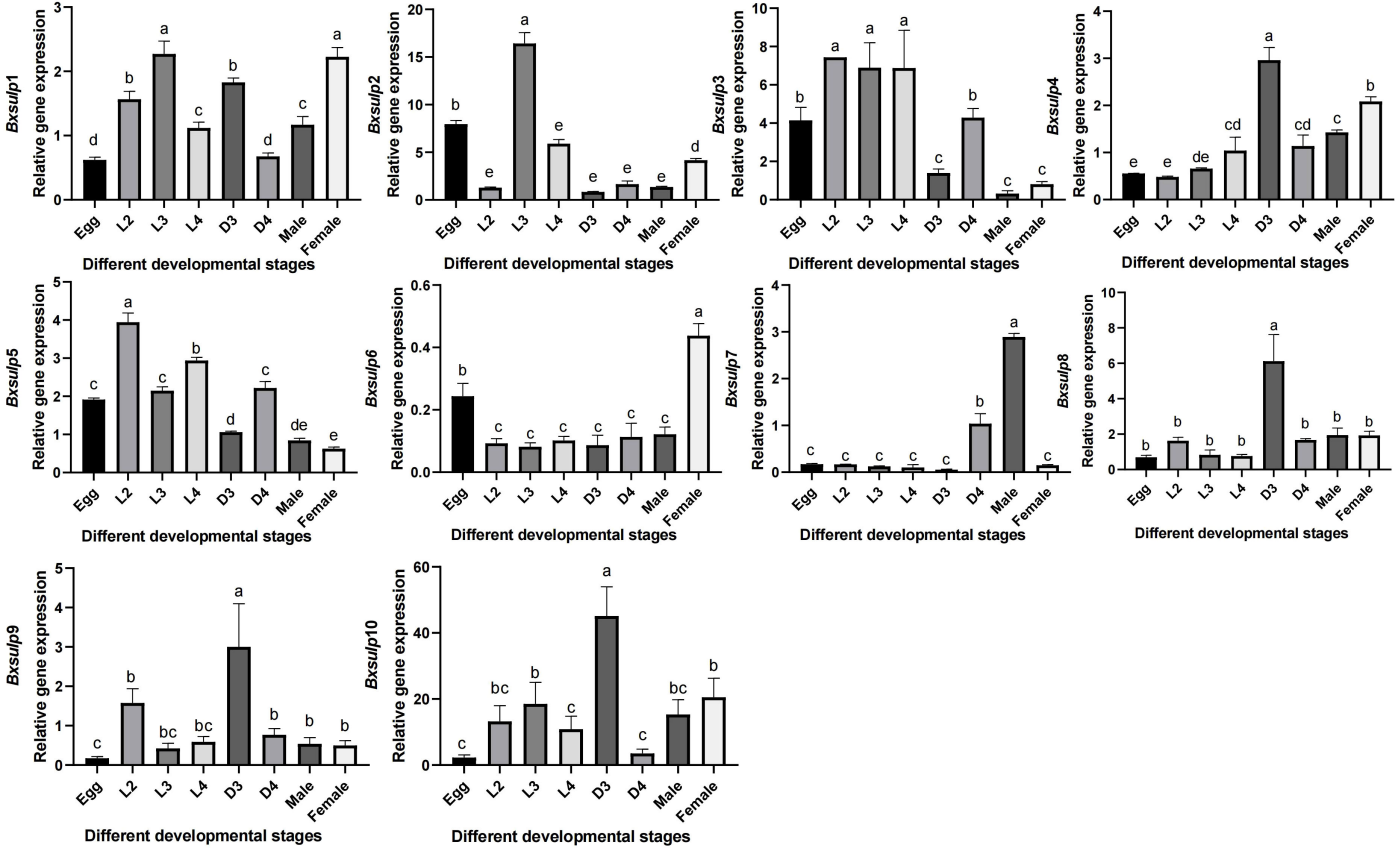
Expression pattern of *Bx-sulp*s in different ages. Egg was egg stage of *B*. *xylophilus*; L2 was second instar larva; L3 was third instar larva; L4 was fourth instar larva; D3 was dauer third-instar larva; D4 was dauer fourth-instar larva; Male was male adult of *B*. *xylophilus*; Female was female adult of *B*. *xylophilus*.

To analyze gene expression in different developmental stages of *B. xylophilus*, we employed reverse-transcription quantitative polymerase chain reaction (RT-qPCR). By analyzing gene expression data from *B. xylophilus* under various nematode states and stress conditions, the expression data of 10 *Bx-sulp*s were obtained. Significant variations in *Bx-sulp*s expression across different nematode states (**Figure 6**). Specifically, *Bx-sulp*s exhibited low expression levels during egg incubation and fourth instar larva (L4). Only two genes, *Bx-sulp*3 and *Bx-sulp*5, were highly expressed in second instar larva (L2). *Bx-sulp*1, *Bx-sulp*2 and *Bx-sulp*3 were highly expressed in third instar larva (L3). Notably, *Bx-sulp*4, *Bx-sulp*8, *Bx-sulp*9, and *Bx-sulp*10 showed heightened expression in dauer third-instar larva (D3), crucial for maintaining essential physiological functions during diapause and enhancing resistance. The overexpression of *Bx-sulp*s potentially bolstered resistance during diapause, with sulfate likely playing a role in cell membrane stabilization and enhancing cell tolerance to extreme conditions. Moreover, *Bx-sulp*1, *Bx-sulp*6 and *Bx-sulp*7 were prominently expressed in female adult nematodes and male adult nematodes, suggesting involvement in sex differentiation. While L2, L3, and L4 stages exhibited similar *sulp* genes expression patterns, the transition to adulthood, either male or female, was marked by significant changes in gene expression profiles.

## 4 Discussion

In recent years, an increasing number of researchers had been investigating the molecular pathways of *B. xylophilus* by RNA-sequence analysis (Zhang et al., 2024). RNA-sequence analysis had primarily been employed to elucidate the pathogenicity, stress resistance, growth, and detoxification mechanisms of *B. xylophilus* (An et al., 2023; Wang et al., 2024). For instance, Li conducted a comparative transcriptomic analysis to investigate the molecular interactions between the *B. xylophilus* and *β*-pinene, a secondary metabolite produced by pine as a defense against nematode infection (Li et al., 2020). The study revealed that a significant proportion of the differentially expressed genes were associated with cytochrome P450 (CYP), UDP-glucuronide transferase (UDT), and short-chain dehydrogenase (SDR) genes. Similarly, Lu analyzed transcriptome data of *B. xylophilus* following exposure to emamectin benzoate (Lu et al., 2020a). The analysis indicated that genes encoding pectate lyases and *β*-1,4-endoglucanases were down-regulated, while those related to glutamate-gated chloride channels (GluCls), *γ*-aminobutyric acid type *β* receptors (GABAB), and ATP-binding cassette transporters (ABC) were upregulated. These genes played crucial roles in the embryonic and larval development, reproduction, as well as the nervous and motor systems of *B. xylophilus*, making them potential targets for *B. xylophilus* control strategies. Further exploration of specific gene families within *B. xylophilus* will offer a solid theoretical foundation for the development of targeted control measures. This study provides theoretical basis for developing control strategies based on *B. xylophilus* by studying BxSUIPs.

The sulfate permease (SULP) family, commonly associated with sulfate transporters (SULTR) which is prevalent in plants and certain microorganisms. SULTR played a crucial role in sulfate absorption and transportation, serving as a carrier protein essential for active sulfate transport in plants (Chen et al., 2024). The SULTR protein comprises 12 membrane-spanning domains (TMDs) at the N-terminal, a sulfur_transp domain at the N-terminal, and a STAS domain at the C-terminal region. These domains are directly involved in sulfate binding, transmembrane transport, and the maintenance of SULTR protein activity and stability (Zhou et al., 2024). In *Heterodera glycines*, an SULTR-type sulphate transporter (HgSULTR1;2) was transcriptionally up-regulated 5- to 12-fold within 24 h of root invasion and RNAi-mediated knock-down reduced both cyst formation and egg hatching by >60%, demonstrating that efficient sulphate uptake is essential for parasitic success (Ding et al., 2016). Sulphate limitation triggers a global stress response in nematodes. In *Caenorhabditis elegans*, sulphate starvation induced dauer formation, implying that precise control of sulphate availability via SULP transporters can modulate developmental decisions and reproduction in *B. xylophilus* (Hacham et al., 2025). In other plant pathogens and symbionts, disruption of sulphate permeases reduced virulence. For example, deletion of the sulphate permease gene *cysP* in the bacterium *Xanthomonas campestris* attenuated growth in plant (Zhang et al., 2016). These findings suggested that *sulp* genes were evolutionarily conserved and functionally diversified across taxa.

Cysteine and methionine synthesis depend on a continuous supply of sulfate; any modulation of SULP-mediated transport could directly impact nematode protein metabolism. As a sulfur-containing compound, ergothioneine can significantly prolong the lifespan of *C. elegans*, improve its mobility and resistance to adverse environments (Petrovic et al., 2025). The diapause stage is a survival strategy of *B. xylophilus* to endure unfavorable conditions like low temperatures and drought, allowing it to remain active across seasons and environments, thereby expanding its transmission range (Vlaar et al., 2021). The high expression of *Bx-sulp*s in the dauer third-instar larva (D3) of *B. xylophilus* indicated its importance in maintaining essential physiological functions and enhancing resistance during diapause, aligning with the notion that sulfur compounds can enhance nematode resilience to adverse environments. In the D3 stage, sulfate likely aids in stabilizing cell membranes and increasing cell tolerance to extreme conditions (Chang et al., 2019). Moreover, the significant expression of *Bx-sulp*6 and *Bx-sulp*7 in female adult nematodes (FM) and male adult nematodes (M) suggested their involvement in sex differentiation, underscoring the potential role of sulfate transport in reproductive processes with implications for nematode population dynamics and pathogenicity. The varying expression patterns in different developmental stages emphasized the significance of elucidating the regulatory mechanisms that control *Bx-sulp*s gene expression, this could facilitate the design of precise interventions to disrupt the nematode’s sulfate transport mechanisms. Future functional studies, including RNA interference (RNAi) and CRISPR-based gene editing, were warranted to dissect the individual contributions of each *Bx-sulp* genes to nematode development and pathogenicity.

The phylogenetic analysis displayed distinct clustering patterns, indicating that BxSULPs form well-supported clades with homologs from other nematodes, suggesting significant sequence conservation and shared ancestry. This observation implied that these proteins likely preserve essential functional features crucial for sulfate transport (Marietou et al., 2018). Conserved motifs were identified across the 10 BxSULPs, associated with key functions such as sulfate binding, transmembrane transport, and protein stability, further supporting their conserved functionality. The chromosomal localization of the 10 *Bx-sulp*s revealed their dispersed distribution across five chromosomes. This dispersion had implications for gene regulation, genes situated on different chromosomes might be subject to diverse regulatory mechanisms and environmental influences (Goeke and Binder, 2024). The non-clustered distribution of *Bx-sulp*s suggested a complex regulatory landscape, possibly involving diverse transcription factors and signaling pathways (Goronzy, 2025). This intricate regulatory network might enable the nematode to finely regulate its sulfate transport mechanisms in response to varying environmental conditions and developmental stages (Rinu et al., 2020). The topological analysis indicated that BxSULP2, BxSULP3, BxSULP4, BxSULP6, BxSULP7, BxSULP8, and BxSULP10 were transmembrane proteins primarily facilitating sulfate transmembrane transport. In contrast, BxSULP1, BxSULP5, and BxSULP9 were intra-membrane proteins that might be secreted outside the cell through signaling peptides, localized within the extracellular matrix, or participate in intercellular signaling upon secretion. The hydropathicity analysis indicated that BxSULP5 and BxSULP9 exhibited hydrophilic properties, which can involve in extractable interactions or signaling processes, consistent with the results of topology analysis. The three-dimensional structural analysis of BxSULPs revealed that their transmembrane segments were primarily composed of *α*-helices. These *α*-helical structures facilitated protein integration into the cell membrane, crucial for forming functional channel configurations that facilitate efficient sulfate ion transport (Wang et al., 2021). These findings provided a structural foundation for comprehending the function and regulation of BxSULPs.

The identification and comparative analysis of BxSULPs represent a significant advancement in comprehending the adaptive strategies of nematodes and their pathogenic interactions with pine hosts. This research elucidates the roles of the BxSULPs in the development and survival, enhancing the understanding of the molecular mechanisms governing development and stress responses of *B. xylophilus*. Disrupting the sulfate transport mechanisms of *B. xylophilus* can potentially impede its ability to thrive within the pine host environment, thereby mitigating its pathogenicity and spread. Furthermore, this study serves as a foundation for future investigations into the gene family of *B. xylophilus* and gene knockout experiments involving the SULP family.

## 5 Conclusions

In conclusion, this study represents the first comprehensive identification and characterization of the complete SULP gene family in *B. xylophilus*. The BxSULPs exhibit highly conserved structural features, indicative of evolutionary stability within nematodes, while minimal structural divergence among them suggests functional consistency and potential synergistic interactions. Expression profiling revealed that *Bx-sulp*4, *Bx-sulp*8, *Bx-sulp*9, and *Bx-sulp*10 were specifically up-regulated during the dauer (D3) stage, implicating these transporters in diapause maintenance and stress adaptation. Conversely, *Bx-sulp*1, *Bx-sulp*6 and *Bx-sulp*7 showed elevated expression in adult males or females, indicating a putative role in reproductive processes and sexual differentiation. The non-clustered genomic distribution and diverse membrane topologies of BxSULPs further provide a structural foundation for their stage-specific and environmentally modulated regulation. Collectively, these findings position the BxSULPs as promising molecular targets for RNAi-based or small-molecule intervention strategies aimed at disrupting critical biological processes such as diapause and fecundity, thereby offering novel prospects for the control of PWD. However, further investigation is required to elucidate the involvement of BxSULPs in the immune mechanisms of *B. xylophilus*. This study establishes a foundational framework for future functional analyses of BxSULPs and identifies potential targets for the development of targeted management strategies against *B. xylophilus* infestations.

## Data Availability

The raw sequencing data BXSULP1-10 generated from genes analyses in this study have been deposited in the NCBI database (PX242223-32).
