# Supplementary material for: Analysis of the sulfate permease family in Bursaphelenchus xylophilus in the nematode development and stress adaptation
Source: Front Plant Sci. 2025 Oct 13;16:1630288. doi: 10.3389/fpls.2025.1630288 (PMC12555069; doi:10.3389/fpls.2025.1630288)
Supplement: Supplementary file 1 [file DataSheet1.pdf]

Supplementary Material

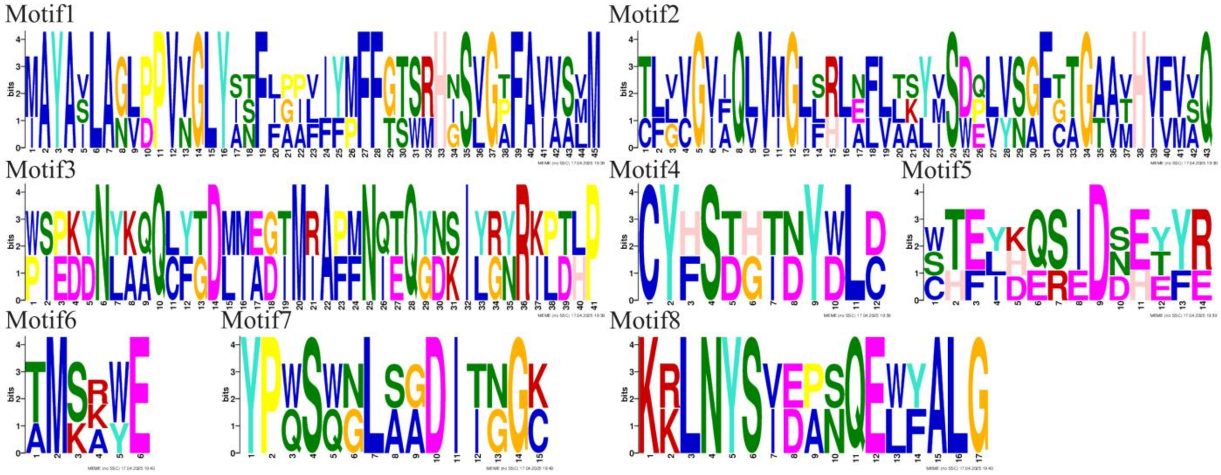

Figure S1. Conserved motif structures of BxSULPs.

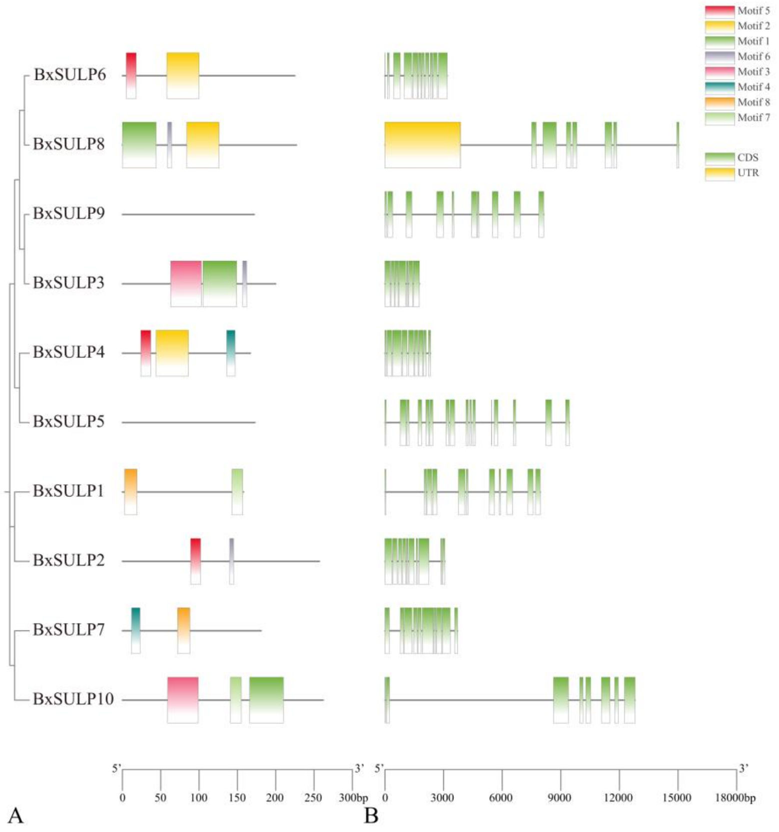

Figure S2. Comparison of the structure motifs distribution across the 10 BxSULPs. Note: A. Distribution of conserved motifs across the 10 BxSULPs. B. Exon-intron structure of *Bx-sulp*s.
